# Visceral adiposity loss is associated with improvement in cardiometabolic markers: findings from a dietary intervention study

**DOI:** 10.3389/fendo.2025.1576599

**Published:** 2025-06-04

**Authors:** Shazana Rifham Abdullah, Ahmad Kamil Nur Zati Iwani, Liyana Ahmad Zamri, Ruziana Mona Wan Mohd Zin, Norhashimah Abu Seman, Nur Azlin Zainal Abidin, Siti Sarah Hamzah, Nur Hayati Azizul, Azahadi Omar, Zamtira Seman, Abqariyah Yahya, Mohd Fairulnizal Md Noh

**Affiliations:** ^1^ Institute for Medical Research, National Institutes of Health, Ministry of Health Malaysia, Selangor, Shah Alam, Malaysia; ^2^ Sector for Biostatistics and Data Repository, National Institutes of Health, Ministry of Health Malaysia, Selangor, Shah Alam, Malaysia; ^3^ Department of Social and Preventive Medicine, Faculty of Medicine, Universiti Malaya, Kuala Lumpur, Malaysia

**Keywords:** visceral fat area, visceral adiposity, cardiometabolic markers, lipid profiles, insulin resistance, intermittent fasting

## Abstract

**Background:**

Visceral adiposity is closely linked with cardiometabolic disorders, but evidence on the extent of visceral fat loss required for significant improvement in cardiometabolic markers remains limited. This study aims to investigate the association between visceral fat area (VFA) reductions and improvements in cardiometabolic markers following a 3-month dietary intervention.

**Methods:**

A total of 175 adults with overweight and obesity were involved in this non-randomized controlled trial. Data on sociodemographic, anthropometric, body composition and biochemistry were collected at baseline and after 3 months of intervention. The multiple logistic regression analysis was conducted to determine the association between VFA loss (no loss, < 5% loss, and ≥ 5% loss) and improvement in cardiometabolic markers. For each cardiometabolic marker, an improvement surpassing the minimum threshold of the third tertile was classified as a good improvement.

**Results:**

Compared to those with no VFA loss, participants with VFA loss of ≥ 5% were significantly associated with a higher improvement in waist circumference (OR 2.97, 95% CI 1.16-7.64), high-density lipoprotein cholesterol (HDL-C) (OR 4.19, 95% CI 1.58-11.14), triglycerides (OR 3.01, 95% CI 1.14-7.92), and glycated hemoglobin (HbA1C) (OR 2.95, 95% CI 1.12-7.79). Other than that, those with < 5% VFA loss were 3.6 times more likely to have a higher improvement in HDL-C compared to those with no VFA loss (OR 4.08, 95% CI 1.36-12.22).

**Conclusion:**

This study found that the magnitude of VFA loss is an independent determinant of improvements in cardiometabolic markers and should be set as a clear target when designing obesity prevention programs.

## 1 Introduction

Over the past two decades, there has been an increase in the prevalence of cardiovascular diseases (CVD) and metabolic conditions such as hypertension (1), type 2 diabetes (2), hyperlipidemia (3), ischemic heart disease (4), and metabolic syndrome (5). While mortality rates from CVD showed a decreasing trend in high-income countries, recent evidence indicates a potential plateau in these declines (6). As a result, CVDs remain the leading cause of death and early morbidity worldwide (6), which is responsible for nearly 30% of all global deaths (7).

Visceral adiposity is strongly associated with cardiometabolic diseases, underscoring relationship between excess visceral fat and increased cardiometabolic risk (8, 9). The Framingham Heart Study indicated a significant positive relationship between abdominal adiposity and CVD risk factors (10). Excess adipose tissue, especially visceral fat, increases insulin resistance by releasing free fatty acids and hormones that disrupt insulin signaling (11), and is also metabolically active, promoting systemic low-grade inflammation. This occurs via the secretion of pro-inflammatory cytokines, which ultimately damage blood vessels and contribute to atherosclerosis, a major risk factor for CVD (12). Visceral adiposity was evaluated using multiple approaches, including imaging techniques such as computed tomography (CT) and dual-energy X-ray absorptiometry (DEXA), both of which are widely regarded as accurate and reliable methods for measuring visceral fat area (VFA). Alongside direct imaging, surrogate markers like waist circumference, waist-to-hip ratio, and waist-to-height ratio were utilized to provide indirect estimates of visceral fat.

Dietary intervention, irrespective of the practices employed, has been demonstrated as a principal strategy for visceral fat reduction (13). Intermittent fasting (IF) is a dietary regime that involves temporal eating restrictions, with popular methods such as time-restricted feeding, the 5:2 diet, and alternate-day fasting (14). Emerging evidence suggests that effective IF regimes “flip” a “metabolic switch” that triggers a series of lipid metabolism and fat mobilization processes (15), which ultimately improves body composition- particularly visceral fat and reduces cardiometabolic risk markers (16, 17). Although studies have shown improvements in visceral fat with IF interventions, data on the effects of 5:2 dry IF on this parameter remain limited. Dry IF, defined as a complete fast without any food or fluid intake (18), is commonly practiced by Muslims during the month of Ramadan.

Since visceral fat is known to contribute to the development of cardiometabolic diseases, reducing it is significantly associated with improvements in cardiometabolic markers such as blood pressure, lipid profiles, glycated hemoglobin (HbA1C), and insulin resistance (19–21). However, the amount of visceral fat reduction needed to achieve these marker improvements is still unclear. Therefore, the primary aim of this study was to determine the extent of VFA loss needed to observe significant improvements in cardiometabolic markers after three months of dietary intervention. Additionally, we examined the association between VFA and cardiometabolic markers in adults with overweight and obesity at baseline.

## 2 Materials and methods

### 2.1 Study design and participants

This study analyzed data from a dietary intervention conducted between 2020 and 2021 among civil servants with overweight and obesity. Details on the study design and protocol have been published elsewhere (16). Briefly, this non-randomized controlled study included a total of 177 participants with overweight or obesity. Participants aged 19 to 59 years with a BMI of ≥23 kg/m² (classified as overweight or obese) who were willing to participate in the intervention (determined through a readiness screening) and provided informed consent were enrolled. The exclusion criteria were as follows: (1) recent involvement in weight loss programs (e.g., intermittent fasting, dietary or physical activity changes aimed at weight reduction); (2) presence of an eating disorder; (3) diagnosis of diabetes, hypertension, or other metabolic conditions (on medication); (4) use of any medications or supplements that could impact study outcomes; (5) pregnancy; and (6) insufficient capacity or language skills to follow the protocol independently.

Participants were divided into two intervention groups: the combined Intermittent Fasting and Healthy Plate (IFHP) group and the Healthy Plate (HP) group. The study had two phases: a supervised phase (first 3 months) and an unsupervised phase (3–6 months). During the supervised phase, participants in the IFHP group practiced dry fasting from dawn to dusk two days a week (Mondays and Thursdays) and followed the HP guidelines on the remaining days. On fasting days, they were encouraged to have a meal before dawn, after which no food or drink was permitted until sunset (approximately 13 hours) (22). Participants in the HP group were instructed to follow the HP guidelines daily, which involved a portion control method where their plate was divided into quarters—one-quarter for protein, one-quarter for complex carbohydrates, and half for fruits and vegetables (23). Although they were encouraged to apply the HP concept to all three main meals per day, adherence was considered met if they applied it to at least one main meal daily. Adherence to dietary protocols was monitored by trained research assistants through a daily photo record of one meal and a weekly fasting log.

Changes in VFA within and between the intervention groups have been reported and discussed in a separate publication (16). A significant reduction in VFA was observed among participants in the IFHP group after 12 weeks, whereas no such change was noted in the HP group. However, as the between-group difference in VFA change was not statistically significant, data from both intervention groups were pooled for the analyses presented in this paper.

This study received approval from the Medical Research and Ethics Committee, Ministry of Health Malaysia (NMRR-19-3261-51726). It was conducted in full compliance with the latest revision of the Declaration of Helsinki and the International Council for Harmonisation Guidelines for Good Clinical Practice. Prior to recruitment, all participants were thoroughly informed about the potential risks of the study, and written informed consent was obtained. This study is registered with ClinicalTrials.gov (NCT05034653).

### 2.2 Anthropometric and body composition assessment

Body weight and height were measured using a Seca electronic scale (Seca GmbH and Co KG) to the nearest 0.1 kg and 0.1 cm, respectively. Participants wore light clothing and removed outer garments and shoes for accurate weight measurement. BMI was calculated as weight (kg) divided by height squared (m²). Waist circumference was measured with a Seca tape (Seca GmbH and Co KG) to the nearest 0.1 cm, at the midpoint between the top of the iliac crest and the lower edge of the last rib, with participants standing. Two measurements were taken for each parameter, and the average was recorded to ensure accuracy.

VFA and other body composition parameters were assessed using an InBody 770 bioimpedance analyzer (BIA, Biospace). Age, height, weight, and sex were entered, and participants, standing barefoot, held the device handles with their thumbs and palms in contact with the electrodes. The device used eight electrodes: two on each thumb, palm, front foot, and back foot. The analysis, which measured body composition using proprietary algorithms built into the device, was completed in approximately two minutes.

### 2.3 Cardiometabolic markers assessment

Participants were required to fast overnight for eight to 10 hours before blood collection. Medical officers collected approximately 15 ml of fasting venous blood from each participant for biochemical tests, including fasting blood glucose, HbA1C, fasting insulin, and a fasting lipid profile [triglycerides, total cholesterol, high-density lipoprotein cholesterol (HDL-C), and low-density lipoprotein cholesterol (LDL-C). Blood samples were processed within two hours, with serum or plasma aliquots stored at −20°C until analysis.

HbA1C was measured by ion exchange high-performance liquid chromatography (Tosoh G8 HPLC analyzer) following National Glycohemoglobin Standardization Program Guidelines. Fasting plasma glucose, triglycerides, total cholesterol, HDL-C, and LDL-C were analyzed using an automated biochemistry analyzer (Dirui CS-400) with reagents from Dirui.

Fasting insulin was measured using Tosoh AIA-360 automated immunoassay analyzer. Insulin resistance was assessed using HOMA-IR (Homeostasis Model Assessment of Insulin Resistance) in this study. HOMA-IR was calculated using fasting insulin and fasting glucose levels. The formula is:


$$\frac{\text{Fasting Insulin }\left(\text{μU}/\text{mL}\right)\text{ x Fasting Glucose }\left(\text{mmol}/\text{L}\right)}{22.5}$$

Blood pressure was measured with an automated upper arm device (Omron Automated Blood Pressure Monitor; HEM 7130). Participants were seated with their arm supported at heart level following 5 minutes of rest. Two measurements were taken, and the average of the readings was calculated to minimize potential measurement errors.

### 2.4 Statistical analyses

Statistical analysis was conducted using the Statistical Package for the Social Science (SPSS) software (version 25; IBM Corp). The Kolmogorov-Smirnov test was used to assess the normality of continuous variables. Data with a normal distribution were presented as mean and standard deviation (SD), while skewed data were summarized as median and interquartile range (IQR).

Logistic regression analysis was conducted to investigate both associations in this study. In the assessment of the relationship between VFA and cardiometabolic markers at baseline, a median VFA of 166 cm² was utilized as the cutoff to classify individuals into low (VFA < 166 cm²) and high (VFA ≥ 166 cm²) categories. For the analysis of the relationship between VFA loss and improvements in cardiometabolic markers, VFA loss was categorized as no loss (VFA gain, no change, or 2% or less loss), less than 5% loss (VFA loss between 2% and 5%), and 5% or more loss. For each cardiometabolic outcome, a change exceeding the minimum value of the third tertile was deemed a significant improvement. All statistical tests were conducted as two-sided, with a significance level established at 0.05.

## 3 Results

### 3.1 Participants’ characteristics

A total of 175 participants (82.9% female, 17.1% male), were included in the analysis. The majority of the participants were Malays (n=139, 79.4%), with a mean age of 34.19 years (SD 7.37). Of the participants, 68.6% (n=120) had secondary education as their highest education level, followed by tertiary (n=32, 18.3%) and primary (n=23, 13.1% (**Table 1**).

**Table 1:** Characteristics of study participants at baseline

Characteristics	Total n=175
**Age (years), mean (SD)**	34.19 (7.37)
Sex, n (%)	
Male	30 (17.1)
Female	145 (82.9)
Ethnicity, n (%)	
Malay	139 (79.4)
Non-Malay	36 (20.6)
Intervention groups, n (%)	
IFHP	91 (52.0)
HP	84 (48.0)
Highest education status, n (%)	
Primary	23 (13.1)
Secondary	120 (68.6)
Tertiary	32 (18.3)
Anthropometric, mean (SD)	
WC (cm)	92.32 (11.07)
VFA (cm^2^)	166.52 (40.44)
Lipid profiles (mmol/L)	
TC, mean (SD)	5.33 (0.98)
LDL-C, mean (SD)	4.02 (0.97)
HDL-C, mean (SD)	1.41 (0.24)
TG (mmol/L), median (IQR)	1.05 (0.78)
Metabolic	
Insulin (mIU/L), median (IQR)	11.00 (9.00)
HOMA-IR, median (IQR)	2.45 (2.08)
FPG (mmol/L), median (IQR)	4.94 (0.57)
HbA1c (%), median (IQR)	5.50 (0.50)
Blood pressure (mmHg)	
SBP, mean (SD)	114.93 (13.29)
DBP, mean (SD)	79.38 (13.00)
**Physical activity (MET-min/week), median (IQR)**	636.00 (726.00)

DBP, Diastolic blood pressure; FPG, Fasting plasma glucose; HbA1C, Glycated haemoglobin; HDL-C, High-density lipoprotein cholesterol; HOMA-IR, Homeostatic Model Assessment for Insulin Resistance; HP, Healthy Plate; IFHP, Intermittent Fasting Healthy Plate; IQR, Interquartile Range; LDL-C, Low-density lipoprotein cholesterol; MET, Metabolic equivalent of task; SBP, Systolic blood pressure; SD, Standard deviation; TC, Total cholesterol; TG, Triglyceride; VFA, Visceral fat area; WC, Waist circumference. Bolded values indicate variable names.

### 3.2 Association between VFA and cardiometabolic markers at baseline

**Table 2** presents the results of simple and multiple logistic regression analyses exploring the relationship between VFA and cardiometabolic markers. Model 1 is unadjusted, while Model 2 controlled for age, race, sex, intervention group, highest education level, and physical activity. Significant associations were identified between VFA and waist circumference, HDL-C, fasting insulin, fasting plasma glucose, and HOMA-IR. Participants with high VFA had significantly higher odds of having a larger waist circumference compared to those with low VFA [Adjusted odds ratio (AOR) 1.30, 95% CI 1.20-1.41]. Furthermore, participants in the high VFA group were 1.1, 2.2, and 1.4 times more likely to exhibit elevated levels of fasting insulin (AOR 1.10, 95% CI 1.04-1.17), fasting plasma glucose (AOR 2.21, 95% CI 1.04-4.67), and HOMA-IR (AOR 1.42, 95% CI 1.15-1.77), respectively, compared to those in the low VFA group. Moreover, those with high VFA were 92% less likely to have elevated HDL-C levels than those with low VFA (AOR 0.08, 95% CI 0.02-0.40) (**Table 2**).

**Table 2:** Association between VFA and cardiometabolic markers at baseline.

VFA	Model 1^a^ OR (95% CI)	Model 2^b^ AOR (95% CI)
Waist circumference		
Low VFA	Reference	
High VFA	1.12 (1.08-1.17)**	1.30 (1.20-1.41)**
Total cholesterol		
Low VFA	Reference	
High VFA	0.95 (0.70-1.29)	1.00 (0.71-1.42)
LDL-C		
Low VFA	Reference	
High VFA	1.04 (0.77-1.41)	1.19 (0.83-1.69)
HDL-C		
Low VFA	Reference	
High VFA	0.32 (0.09-1.15)	0.08 (0.02-0.40)*
Triglycerides		
Low VFA	Reference	
High VFA	0.75 (0.48-1.15)	1.16 (0.70-1.93)
Fasting insulin		
Low VFA	Reference	
High VFA	1.09 (1.04-1.15)**	1.10 (1.04-1.17)**
Fasting plasma glucose		
Low VFA	Reference	
High VFA	1.79 (1.02-3.15)*	2.21 (1.04-4.67)*
HbA1c		
Low VFA	Reference	
High VFA	1.51 (0.90-2.52)	1.50 (0.85-2.84)
HOMA-IR		
Low VFA	Reference	
High VFA	1.36 (1.12-1.66)*	1.42 (1.15-1.77)*
Systolic blood pressure		
Low VFA	Reference	
High VFA	1.00 (0.98-1.03)	1.03 (1.00-1.06)
Diastolic blood pressure		
Low VFA	Reference	
High VFA	1.01 (0.98-1.04)	1.03 (0.99-1.07)

*p<0.05, **p<0.001.

a Unadjusted model.

b Adjusted for age, sex (male or female), race (Malay, or non-Malay), intervention group (IFHP or HP), highest education status (primary, secondary or tertiary), and physical activity (MET-min/week).

95% CI, 95% confidence interval; AOR, Adjusted odds ratio; HbA1C, Glycated haemoglobin; HDL-C, High-density lipoprotein cholesterol; HOMA-IR, Homeostatic Model Assessment for Insulin Resistance; LDL-C, Low-density lipoprotein cholesterol; OR, Odds ratio; VFA, Visceral fat area.

### 3.3 Association between VFA changes and improvement in cardiometabolic markers after three months

**Table 3** presents the results of stepwise logistic regression analysis, assessing how different categories of VFA reduction impact improvements in cardiometabolic markers after three months. Model 1 is unadjusted, Model 2 adjusts for age, race, sex, intervention group, and highest education level, while Model 3 includes additional adjustments for physical activity.

**Table 3:** Association between VFA changes and improvement in cardiometabolic parameters after three months.

Improvement in cardiometabolic markers	Model 1^a^ OR (95% CI)	Model 2^b^ AOR (95% CI)	Model 3^c^ AOR (95% CI)
Waist circumference			
No VFA loss	Reference		
< 5% VFA loss	1.16 (0.40-3.40)	0.84 (0.27-2.62)	0.76 (0.24-2.39)
≥ 5% VFA loss	3.69 (1.59-8.56)*	3.60 (1.45-8.94)*	2.97 (1.16-7.64)*
Total cholesterol			
No VFA loss	Reference		
< 5% VFA loss	0.41 (0.13-1.31)	0.44 (0.13-1.49)	0.44 (0.13-1.48)
≥ 5% VFA loss	1.08 (0.47-2.46)	1.23 (0.51-3.01)	1.20 (0.48-3.00)
LDL-C			
No VFA loss	Reference		
< 5% VFA loss	0.95 (0.35-2.62)	1.07 (0.37-3.06)	1.04 (0.36-2.99)
≥ 5% VFA loss	1.06 (0.46-2.47)	1.00 (0.41-2.42)	0.92 (0.37-2.31)
HDL-C			
No VFA loss	Reference		
< 5% VFA loss	3.65 (1.35-9.85)*	3.84 (1.30-11.34)*	4.08 (1.36-12.22)*
≥ 5% VFA loss	3.44 (1.47-8.08)*	3.60 (1.41-9.19)*	4.19 (1.58-11.14)*
Triglycerides			
No VFA loss	Reference		
< 5% VFA loss	2.15 (0.80-5.81)	2.09 (0.72-6.11)	2.26 (0.76-6.69)
≥ 5% VFA loss	2.33 (1.00-5.43)	2.56 (1.01-6.50)*	3.01 (1.14-7.92)*
Fasting insulin			
No VFA loss	Reference		
< 5% VFA loss	0.60 (0.20-1.81)	0.46 (0.14-1.51)	0.46 (0.14-1.49)
≥ 5% VFA loss	1.53 (0.68-3.46)	1.55 (0.63-3.80)	1.47 (0.58-3.71)
Fasting plasma glucose			
No VFA loss	Reference		
< 5% VFA loss	0.64 (0.21-1.92)	0.67 (0.20-2.30)	0.68 (0.20-2.34)
≥ 5% VFA loss	1.62 (0.71-3.68)	1.80 (0.68-4.74)	1.88 (0.69-5.13)
HbA1C			
No VFA loss	Reference		
< 5% VFA loss	1.21 (0.43-3.39)	0.96 (0.33-2.86)	1.10 (0.36-3.83)
≥ 5% VFA loss	1.90 (0.82-4.41)	2.25 (0.89-5.66)	2.95 (1.12-7.79)*
HOMA-IR			
No VFA loss	Reference		
< 5% VFA loss	0.60 (0.20-1.81)	0.64 (0.20-2.11)	0.64 (0.19-2.09)
≥ 5% VFA loss	1.53 (0.68-3.46)	1.84 (0.72-4.70)	1.78 (0.68-4.69)
Systolic blood pressure			
No VFA loss	Reference		
< 5% VFA loss	1.91 (0.73-5.02)	1.69 (0.62-4.64)	1.76 (0.64-4.87)
≥ 5% VFA loss	1.05 (0.45-2.48)	1.02 (0.41-2.53)	1.13 (0.44-2.87)
Diastolic blood pressure			
No VFA loss	Reference		
< 5% VFA loss	1.23 (0.46-3.32)	1.31 (0.45-3.84)	1.35 (0.46-3.97)
≥ 5% VFA loss	1.13 (0.49-2.62)	1.79 (0.70-4.56)	1.89 (0.73-4.92)

*p<0.05.

a Model 1: Unadjusted model.

b Model 2: Adjusted for age, sex (male or female), race (malay or non-malay), intervention group (IFHP or HP) and highest education status (primary, secondary or tertiary).

c Model 3: Additional adjustment for physical activity (MET-min/week).

95% CI, 95% Confidence Interval; AOR, Adjusted odds ratio; DBP, Diastolic blood pressure; FPG, Fasting plasma glucose; HbA1C, Glycated haemoglobin; HDL-C, High-density lipoprotein cholesterol; HOMA-IR, Homeostatic Model Assessment for Insulin Resistance; LDL-C, Low-density lipoprotein cholesterol; OR, Odds ratio; SBP, Systolic blood pressure; VFA, Visceral fat area.

A significant association was observed between waist circumference improvement and ≥5% VFA loss across all three models. While the strength of this association slightly decreased after adjusting for confounding factors, it remained significant. Participants with a VFA loss of 5% or more were 3 times more likely to experience high improvement in waist circumference compared to those with no VFA loss (AOR 2.97, 95% CI 1.16-7.64).

In contrast, the strongest associations were found in the final models linking HDL-C improvement to both VFA loss categories (<5% and ≥5% VFA loss). Participants with a VFA loss of <5% had 4.1 times higher odds of achieving significant HDL-C improvement (AOR 4.08, 95% CI 1.36-12.22), while those with a VFA loss of ≥5% had 4.2 times higher odds (AOR 4.19, 95% CI 1.58-11.14), compared to those with no VFA loss.

A significant association between triglyceride improvement and a VFA loss of 5% or more was observed only after adjusting for confounders, and the association became stronger following additional adjustments for physical activity in Model 3. Participants with a VFA loss of ≥5% were 3 times more likely to achieve high triglyceride improvement compared to those with no VFA loss (AOR 3.01, 95% CI 1.14-7.92).

The association between a VFA loss of ≥5% and HbA1C improvement became significant only in Model 3, after adjusting for all confounders. Participants with a VFA loss of ≥5% had 3.0 times higher odds of having significant HbA1C improvement after 3 months compared to those with no VFA loss (AOR 2.95, 95% CI 1.12-7.79). However, no significant associations were found between VFA changes and improvements in total cholesterol, LDL-C, fasting insulin, fasting plasma glucose, HOMA-IR, systolic blood pressure, or diastolic blood pressure (**Table 3**).

## 4 Discussion

Our study explored the relationships between VFA and various cardiometabolic markers, including waist circumference, lipid profiles, glucose parameters, insulin resistance, and blood pressure, both at baseline and after three months. At baseline, VFA was significantly associated with waist circumference, HDL-C, fasting insulin, fasting plasma glucose, and HOMA-IR. Following three months of dietary interventions, a VFA loss of 5% or more was significantly linked to greater improvements in waist circumference, HDL-C, triglycerides, and HbA1C. Additionally, even a VFA reduction of less than 5% showed a significant association with higher improvements in HDL-C.

There was a major gender disparity among participants in our study (82.9% female vs. 17.1% male), which could be attributed to several factors. Women are often subjected to greater pressure regarding body image and are more likely to internalize weight-related stigma, motivating them to lose weight through obesity prevention programs. Additionally, women tend to visit healthcare providers more frequently than men, increasing their healthcare interactions and facilitating participation in such programs. In contrast, men often downplay weight-related health concerns. They are less likely to perceive themselves as overweight or at risk, even when they meet the criteria for obesity, leading to lower engagement in weight management programs (24, 25). According to the 6th Edition Clinical Practice Guideline Management of Dyslipidemia 2023, the triglycerides and HDL-C levels of our participants were within normal range, while the mean total cholesterol and LDL-C levels exceeded the recommended cut-off values of > 5.2 mmol/L and >3.0 mmol/L, respectively (26). Blood pressure measurements of the participants were within the normal values (27).

Waist circumference is widely recognized as a useful and practical indicator of visceral obesity (28), with numerous studies demonstrating a strong correlation between the two (29, 30), confirming the finding of our study. Although waist-to-hip ratio (WHR) was initially favored as an indicator of abdominal obesity and linked with increased risk of cardiometabolic diseases (31), later evidence indicated that waist circumference alone has a stronger association with the absolute amount of intra-abdominal or visceral fat - the fat depot that poses the greatest health risk (32, 33).

Our study found that HDL-C was significantly associated with VFA, aligned with the findings from previous studies (34–36). In contrast to subcutaneous fat, visceral adipose tissues are metabolically active and sensitive to lipolysis, which can affect HDL-C directly and indirectly. The lipolysis of white adipose tissue releases large amounts of free fatty acids (FFAs) into the bloodstream, causing an influx of FFAs to the liver via the portal vein and stimulate the hepatic synthesis and production of very-low-density lipoprotein (VLDL) (37). These VLDL particles, which are triglyceride-rich lipoprotein, may reduce HDL-C levels by increasing the transfer of triglycerides to HDL, which in turn are hydrolyzed by hepatic lipase (38). Indirectly, elevated FFAs could lead to insulin resistance (39) and modify cholesteryl ester transfer protein (CETP) activity (40), which have significant roles in HDL metabolism. Insulin resistance impairs HDL synthesis and function, while increased CETP activity promotes the transfer of cholesteryl esters from HDL to other lipoproteins, reducing HDL-C levels (38).

The improvement in HDL-C was evident with a reduction in VFA of at least 2% from baseline, achieved within a relatively short period of 12 weeks. Our finding contrasts with a study conducted among postmenopausal Japanese women with obesity who followed the Kagawa Nutrition University Diet, where a significant increase in HDL-C was only observed after 105 weeks (19). A previous study suggested that tissue levels of lipoprotein lipase could decrease by 50-80% during acute caloric restriction (41), supporting our results. Lipoprotein lipase is an enzyme that responsible for the hydrolysis of triglycerides in chylomicron and VLDL, releasing surface components that aid in the formation and maturation of HDL particles. However, our findings need to be interpreted cautiously due to high variability as reflected in the wide confidence interval.

Increased visceral adiposity has been shown to be an independent risk factor for higher triglycerides (42, 43). Excess VFA contributes to fat dysfunction and chronic low-level inflammation, which are key mechanisms underlying dyslipidemia. This dysfunction is partly driven by the secretion of monocyte chemoattract protein-1 (MCP-1) by adipocytes, which induce macrophages infiltration into the adipose tissue and cause the release of inflammatory cytokines such as tumour necrosis factor (TNF)-α and interleukins 6 (44). However, our study showed no baseline association between VFA level and triglycerides, contrasting with earlier studies (34, 45). Interestingly, we discovered that reducing VFA by 5% and more led to significant improvement in triglycerides. The finding aligns with a study investigating the effect of longitudinal changes in VFA on metabolic risk factors in Japanese men (20). Although this study did not demonstrate such effects, the positive changes are likely attributable to the improvement in inflammation following the reduction in visceral adiposity. In obese individuals, the reduction of body fat mass, particularly visceral fat, improves mitochondrial function in adipocytes and lowers cytokines production, playing a vital role in the management of the inflammatory response (46).

A study conducted among Asian Indians found that visceral fat, but not subcutaneous fat, was significantly associated with glucose tolerance and insulin resistance (47), consistent with findings from our study and a recent study among U.S. adults (21). This association may be explained by the direct drainage of free fatty acids through the portal vein, which impairs liver and systemic insulin sensitivity via the secretion of adipose-specific cytokines like leptin and adiponectin, as well as inflammatory markers (48). To elucidate if the differences between subcutaneous and visceral fat in their relationship with glucose metabolism and insulin resistance are due to intrinsic properties and anatomical location, Tran et al. transplanted subcutaneous fat from donor mice into the visceral region of recipient mice and vice versa. Their findings showed a reduction in body weight, total fat mass, glucose levels, and insulin levels in the group with subcutaneous fat transplanted into the visceral cavity (49).

Although no significant association was observed between the level of VFA and HbA1C at baseline, a loss in VFA by 5% or more significantly improved the parameter, which became statistically significant only after adjusting for physical activity in the final model. Physical activity has a well-established impact on enhancing insulin sensitivity, reducing visceral adiposity, and improving glucose metabolism. These effects can directly influence HbA1C levels, independent of reduction in fat (50, 51). The adjustment for physical activity in model 3 isolates the specific effect of visceral fat loss on HbA1C by accounting for the overlapping influence of physical activity on both variables.

As mentioned previously, our intervention consists of intermittent fasting and portion control (16). Fasting and portion control are dietary approaches that have been shown to effectively reduce visceral fat and improve cardiometabolic markers (16, 52). Both strategies adopted calorie restriction concept, which leads to a reduced energy intake and a negative energy balance. This state stimulates lipolysis, preferentially targeting visceral adipose tissue due to its greater metabolic activity. As described by Soeters et al., short-term fasting induces a notable shift in energy substrate utilization, with the body reducing its reliance on carbohydrates and increasing the use of fatty acids for energy (53). Studies on short-term fasting has demonstrated that blood glucose drop while whole-body lipolysis and fat oxidation markedly rise within the first 24 hours of fasting (53, 54). These changes are primarily attributed to a decrease in plasma insulin levels, heightened sympathetic nervous system activity, and elevated growth hormone levels (53). Additionally, fasting and portion control have been associated with favorable changes in lipid profiles, including reduced triglycerides, LDL cholesterol, and increased HDL cholesterol, likely mediated by improved hepatic lipid metabolism and reduced *de novo* lipogenesis (55). These findings suggest that visceral adipose tissue reduction may play a central role in mediating the effects of these dietary interventions on cardiometabolic markers, which warrant further analysis. However, this lies beyond the scope of the present study.

To the best of our knowledge, this is the first study to present VFA changes as percentages and examine their association with improvements in cardiometabolic markers. Other than enhancing interpretability, percentages provide a relative measure that facilitate comparisons across datasets or studies, where initial baselines values may vary significantly (56). Additionally, the experimental design of this study represents the gold standard for evaluating the direct impact of VFA reduction on cardiometabolic health by establishing a strong association while controlling for confounding variables, ensuring robust and reliable results.

There are several limitations to consider in this study. The small number of male participants in our study limits the generalizability of the findings to men. Regarding body composition assessment, this study utilized BIA instead of the gold standard, DEXA. While DEXA is highly precise and ideal for measuring body composition, its application in population studies is often limited by factors such as high cost, time requirement, radiation exposure, and lack of portability. BIA, on the other hand, offers a more practical solution for large-scale and population-based studies because of its portability, speed, non-invasive nature, and cost-effectiveness. Furthermore, research has demonstrated that BIA is valid and reliable for body composition measurement, particularly in population-based studies (57).

While BIA offers several advantages, it is important to recognize its limitations in accurately differentiating between visceral adipose tissue (VAT) and subcutaneous adipose tissue (SAT) when compared to advanced imaging modalities such as computed tomography (CT) and magnetic resonance imaging (MRI) (58). BIA estimates body composition based on the electrical resistance of tissues but lacks the precision necessary to reliably distinguish between VAT and SAT, particularly in individuals with diverse body compositions. Additionally, factors such as hydration status, body size, and the specific BIA device used can affect the accuracy of measurements, further complicating the differentiation of VAT from SAT. Therefore, while BIA remains a valuable tool for general body composition screening, its limitations must be carefully considered when interpreting results, especially in clinical or research contexts requiring precise fat distribution assessments. Nevertheless, BIA continues to be useful in studies with limited resources, such as large-scale epidemiological research and intervention trials, where estimated VAT values can still reveal meaningful trends and associations despite some degree of measurement imprecision. Given that VAT changes were not objectively assessed in this study due to these constraints, we infer that the observed reduction in body fat likely stemmed primarily from VAT (16), due to its higher metabolic activity and pro-inflammatory characteristics (59).

## 5 Conclusions

This study provides compelling evidence of the association between VFA and key cardiometabolic markers, including waist circumference, HDL-C, triglycerides, insulin resistance and glucose parameters. Our findings emphasize the importance of targeting VFA reduction in obesity management programs, as even a modest loss of 5% or more in VFA was linked to significant metabolic benefits. These results highlight the relevance of VFA as a critical target for interventions aimed at improving cardiometabolic health. Future research is needed to validate these findings across diverse populations and to explore the mechanisms underlying the relationship between VFA reduction and cardiometabolic improvements. Such efforts could inform the development of more targeted and effective obesity prevention and management strategies.

## Data Availability

The raw data supporting the conclusions of this article will be made available by the authors, without undue reservation.
